# Safety of Cyproheptadine, an Orexigenic Drug. Analysis of the French National Pharmacovigilance Data-Base and Systematic Review

**DOI:** 10.3389/fped.2021.712413

**Published:** 2021-09-29

**Authors:** Valérie Bertrand, Nathalie Massy, Nancy Vegas, Valérie Gras, Christel Chalouhi, Marie-Pierre Tavolacci, Véronique Abadie

**Affiliations:** ^1^Department of Pediatrics, Le Havre Hospital, Le Havre, France; ^2^Regional Center of Pharmacovigilance, Rouen University Hospital, Rouen, France; ^3^General Pediatrics Unit, Necker University Hospital, Paris, France; ^4^Refferal Center for Rare Disease ≪ Pierre Robin Sequence and Sucking and Swallowing Congenital Disorders ≫, Necker University Hospital, Paris, France; ^5^Regional Center of Pharmacovigilance, Amiens University Hospital, Amiens, France; ^6^CIC104 Rouen University Hospital and INSERM 1073, Rouen, France; ^7^Paris University, Paris, France

**Keywords:** cyproheptadine, adverse (side) effects, appetite, orexigenic, cholestase, liver failure

## Abstract

**Objectives:** Cyproheptadine is a first-generation H1-antihistamine drug first that was distributed in the 1960s. While its orexigenic effect was observed early, cyproheptadine is not yet authorized for this indication in all countries today. There is an increasing medical interest and demand for the orexigenic effect of cyproheptadine, especially in children with poor appetite. As cyproheptadine might be evaluated in future clinical trials, we wanted to assess its safety profile.

**Methods:** Using the French national pharmacovigilance database, we retrospectively analyzed all pediatric and adult reports of adverse effects of cyproheptadine recorded since its first distribution in France. Next, we performed a systematic review of the literature of cyproheptadine adverse effects.

**Results:** Since 1985, 93 adverse effects were reported in the French pharmacovigilance database (adults 81.7%, children 18.3%); these were mainly neurological symptoms (*n* = 38, adults 71%, children 28.9%), and hepatic complications (*n* = 15, adults 86.7%, children 13.3%). In the literature, the most frequent adverse effect reported was drowsiness in adults or children, and five case reports noted liver complications in adults. We estimated the frequency of hepatic adverse effects at 0.27 to 1.4/1000, regardless of age.

**Conclusion:** Cyproheptadine can be considered a safe drug. Mild neurological effects appear to be frequent, and hepatotoxicity is uncommon to rare. Randomized controlled trials are needed to evaluate the safety and efficacy of cyproheptadine before authorization for appetite stimulation, especially in young children as studies at this age are lacking. Possible hepatic complications should be monitored, as very rare cases of liver failure have been reported.

## Introduction

Cyproheptadine (Periactine©) is a first-generation H1-antihistamine drug, that was first distributed in the 1960s. Its indications were acute or chronic allergy and pruritus in dermatologic diseases. Soon after, its effect on appetite stimulation appeared as an interesting side effect (1, 2). In 1994, Canadian authors first questioned this indication as most of the studies supporting this orexigenic effect had major methodologic flaws, and it was finally removed from the official recommendations (3). In France, the last marketing authorization date was December 1997 for “allergic pathologies such as rhinitis, conjunctivitis, urticarial” in adults or children above 6 years old. In the 2000s, cyproheptadine was evaluated again in undernourished children with cancer, cystic fibrosis and Silver-Russell syndrome, with variable but interesting results (4–6). Currently, cyproheptadine is authorized for its orexigenic effect in adults and children above 2 years old in the United States. Cyproheptadine has also been evaluated for functional digestive disorders and migraine prophylaxis (7, 8).

This renewed interest in cyproheptadine is first due to a potentially large medical demand, especially for children with insufficient or very selective appetites, or who need nutritional support. Second, there are no other drugs that can stimulate appetite and food intake without significant adverse effects (AEs). Harrison et al. recently published a systematic analysis of cyproheptadine's efficacy and concluded that “cyproheptadine appears to be a safe, generally well-tolerated medication that has utility in helping facilitate weight gain in patients drawn from a variety of underweight populations” (9). In spite of weak scientific evidence, many patients and parents are currently using cyproheptadine (or asking doctors about it) because of its positive comments on non-scientific websites and its accessibility without prescription as an “over-the-counter drug” (10–12).

First-generation H1-antihistamine drugs are known to have various AEs since H1 receptors are distributed throughout the body. These drugs interact with cerebral nervous system H1, muscarinic, serotonin, and alpha-adrenergic receptors, and interfere with cardiac ions channels. Newer-generation anti-H1 drugs have less central nervous system AEs due to lower concentrations in the brain and have superseded first-generation drugs for allergic indications (13, 14). The majority of AEs described with cyproheptadine are moderate (drowziness, dizziness) (7, 15–17), although rare cases of acute liver failure have been also reported (18). In cases of overdose, cyproheptadine was associated with anticholinergic syndrome, seizures, psychosis, and cardio-respiratory arrests (19).

Because cyproheptadine might be assessed in future clinical trials or used by patients for its orexigenic effect, we wanted to evaluate its safety profile. For this purpose, we collected all pediatric and adult reports of cyproheptadine AEs recorded in the French national pharmacovigilance database since the first distribution of cyproheptadine in France. Next, we performed a systematic (PRISMA-compliant) review of published reports of cyproheptadine AEs.

## Materials and Methods

### Analysis of the French Pharmacovigilance Database

We retrospectively collected and analyzed all reports of AEs involving cyproheptadine exposure, recorded between 1985 and December 31st 2020 in the French pharmacovigilance database (20). Reports were selected by using the drug name “cyproheptadine,” and only reports in which cyproheptadine was “suspected” were kept. For all patients, we recorded anonymously their age, sex, indication for cyproheptadine use, clinical characteristic of the AE, list of concomitant medications, dosage, delay between the first exposure and the occurrence of the AE, and clinical evolution.

To evaluate a potential causal relationship between the drug exposure and the occurrence of an AE, the French pharmacovigilance database uses a score, defined in the 1985 version, based on the evaluation of eight criteria divided into three groups: chronology, semiology and bibliographic data. Once combined, the chronological (C) and semiological (S) scores yield an “intrinsic” causality score ranging from 0 (unlikely) to 4 (very likely) (Table 1) (21). The eighth criterion derives an “extrinsic” or bibliographic score (B) for the reaction from a classification of the available scientific literature.

**Table 1:** French imputability (I) score.

**Chronology (C)**	**Semiology (S)**		
	**S 1**	**S 2**	**S 3**
C 0	I 0	I 0	I 0
C 1	I 1	I 1	I 2
C 2	I 1	I 2	I 3
C 3	I 3	I 3	I 4

*I 0: excluded imputability.*

*I 1: doubtful imputability.*

*I 2: possible imputability.*

*I 3: probable imputability.*

*I 4: very likely imputability*.

To estimate the frequency of these AEs, we tried to determine how many people were exposed to the medication in France during the studied period. Since cyproheptadine is in free sale, we could not use the national social security database. We used data from OpenHealth, a private company specialized in collecting and analyzing health data. OpenHealth collects drugs sales data from approximately half of the retail pharmacies in France. We obtained data for the number of cyproheptadine boxes sold between 2008 and December 31st 2020 in France.

### Literature Systematic Review of Adverse Events of Cyproheptadine

**Figure 1:**
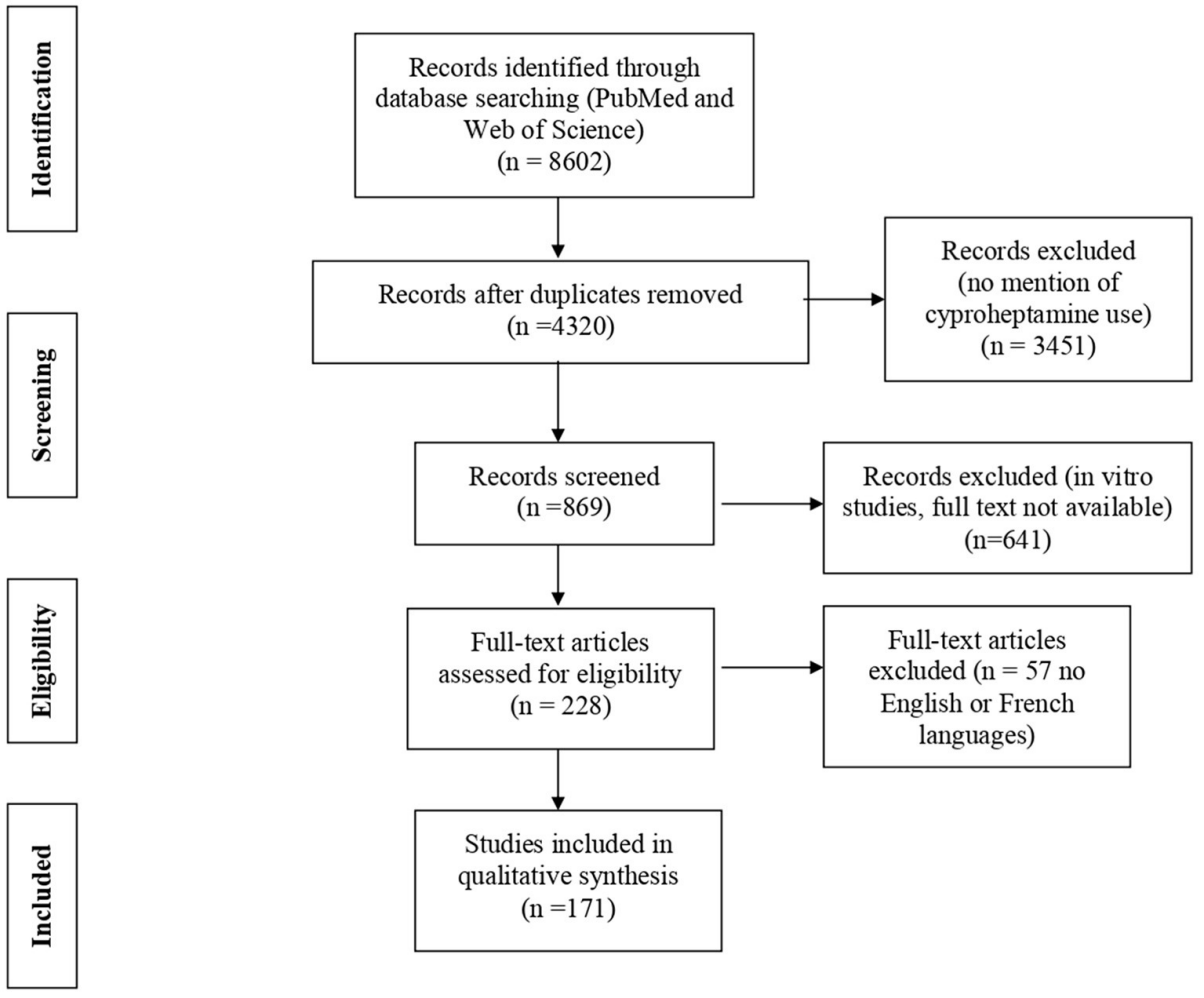
Flow chart of the search strategy for the literature review.

We applied the PRISMA guidelines to perform a systematic review of all the studies of cyproheptadine used as a drug and reporting adverse events. We searched for original articles, case reports, and letters to the editor that reported cases of adverse events with this drug in two electronic databases (PubMed and Web of Science) by using the following keywords (both as free text and MeSH terms): “cyproheptadine,” “adverse effect,” “hepatic,” “review.” Relevant articles were first selected according to their titles. Abstracts and full texts of selected abstracts were reviewed, and references were screened for additional articles. Searches were carried out from 1960 to December 2020. Only articles with full text available in English or French were considered (Figure 1). Descriptive analysis involved frequencies and percentages for qualitative variables and median (range) as appropriate for quantitative variables.

## Results

### Analysis of the French Pharmacovigilance Database

Over the 36 years of the analysis period, a total of 93 AEs were reported in the French pharmacovigilance database (Table 2). The first report dates from 1985 and the last in 2020. Patients with AEs had a median age of 61.5 years (range 2 months to 99 years), and the sex ratio (M/F) was 0.78. Overall, 76 AEs concerned adults, and 17 AEs concerned children (0–18 years old, 58.8% ≤ 4 years old).

**Table 2:** Cyproheptadine adverse effects (AEs) reported to the French national pharmacovigilance database between 1985 and December 31, 2020 (*n* = 93).

**Type of AEs (n)**	**Sexe/age (years)**	**Indication**	**Daily dose (mg)**	**Delay after introduction (days)**	**Concomittant suspect medication**	**CY discontinuation**	**Resolution (duration of follow-up days)**	**Imputability Score**
**Neurologic (38)**								
Drowsiness 7 Drowsiness, amnesia	F93 F1.5 M26 M1 F80 F21 F82	Anorexia breastfeeding Allergy NA NA Urticaria Orexigenic	8 4 4 2 8 8 8	2 30 1 1 1 120 NA	omeprazole, attapulgite, racecatodril N N hydroxyzine N aerius zolpidem, clomipramine	Y Y Y NA Y Y NA	Y (3) Y (NA) Y (2) NA (NA) Y (NA) NA Y (NA)	C2S1B3I1 C2S2B3I2 C2S1B3I1 C2S2B3I2 C2S1B3I1 C1S1B3I1 C2S1B3I1
Confusion 6	M73 M92 F64 F89 M60 F75	NA NA NA NA NA NA	4 NA NA 12 8 NA	3 3 NA 6 4 48	N N N quinine benzoate amoxicillin, ranitidine, morphine clotiazepam, venlafaxine	Y Y Y NA NA Y	Y (NA) Y (NA) Y (NA) Y (NA) Death (UR) Y (60)	C2S2B2I2 C2S2I2 C2S1B3I1 C2S2B3I2 C1S1B0I1 C2S2B3I2
Seizures 5	M4 F39 M76 M30 M2months	Accident NA NA NA breastfeeding	NA 12 12 NA 12	1 15 16 25 60	N buspirone, heptaminol, maprotinil, PCT-caffeine ornithine, clomipramine, PCT-codeine hydroxyzine, calcifediol N	Y NA NA Y Y	Y (1.5) Y (NA) Death (UR) Y (NA) Y(NA)	C1S1B3I1 C2S1B2I1 C2S1B2I1 C2S2B2I2 C1S1B3I1
Agitation 5	F3 F86 F20 M2 F30	NA NA Anorexia NA Anorexia	8 12 8 2 4	4 12 2 8 180	triprolidine N N vitamins solution N	Y Y Y NA Y	Y (1) Y (NA) Y (NA) Y (NA) Y (NA)	C2S2B3I2 C2S1B3I1 C2S1B3I1 C2S1B2I1 C2S1B3I1
Hallucination 5	M73 F15 M92 F90 F28	NA Suicide attempt Anorexia NA Suicide attempt	12 96 12 NA 8	9 NA 4 5 1	N N N N N	Y Y Y Y Y	Y (2) Y (NA) Y (NA) Y (NA) Y (NA)	C2S2B3I2 C2S3B3I3 C1S1B3I1 C2S3B3I3 C2S2B3I2
Asthenia 2	F89 F20	NA Allergy	12 4	12 NA	N N	NA Y	Y (NA) Y (NA)	C2S1B3I1 C1S1B3I1
Paresthesia 2	M68 M12	NA Anorexia	4 4	NA 4	Dihydroergotamine N	NA Y	Y (NA) Y (NA)	C1S1B3I1 C1S1B1I1
Delirium 1 HTIC 1 Facial paralysis 1 Trembling + dry mouth 1 Choreoathetosis 1 Dyskinesia 1	M99 M4.5 F2 F38 F10 F91	NA NA Allergy Anorexia NA NA	4 20 4 4 4 4	9 21 3 1 10 45	N vitamins solution niaprazine N N paroxetine	Y Y NA Y NA Y	Y (NA) Y (10) NA (NA) Y (1) Y (NA) Y (NA)	C1S1B3I1 C2S1B1I1 C2S1B0I1 C1S3B3I2 C2S1B3I1 C2S2B1I2
**Hepatic (15)**								
Acute hepatic failure 3 Moderate cytolysis 7 Cholestasis Cholestatic hepatitis Cholestatic hepatitis + renal failure High SAP level High GGT level	M78 M1 F4.5 F94 F25 M67 F75 F24 F82 M36 F26 M71 F38 F44 M75	NA Orexigenic NA NA NA NA NA NA NA NA NA NA Orexigenic NA NA	12 4 NA 8 4 NA 12 NA A NA NA NA NA 12 12	30 30 NA 6 15 60 15 10 11 2 NA 30 NA NA 5	N N fenofibrate, rifampicin, dimethicone, isoniazide naftidrofuryl, buflomedil, nicardipine, furosemide diosmin, tranexamic acid, rabeprazole bosentan, tadalafil PCT, triazolam N PCT-DXP, calcitonin haloperidol, levomepromazine, loxapine, methylphenidate, diazepam, buprenorphine cyamemazine, clonidine, alimemazine, clorazepate isoniazid, rifampicin, ranitidine flunitrazepam, folic acid, sulfaguanidine, nifuroxazide, loperamide levonorgestrel-EE, prazepam, PCT, paroxetine theophylline, PCT-DXP	Y Y NA NA Y Y Y Y Y N NA NA NA NA NA	Y (NA) Death (syphilis) NA (Hepatitis B) Y (NA) Y (15) Y (NA) Y (7) NA (NA) N (NA) Y (NA) Y,Hepatitis C (NA) Y (NA) Y (240) NA (NA) NA (NA)	C2S1B2I1 C1S1B1I1 C1S1B2I1 C2S1B2I1 C2S2B3I2 C2S1B1I1 C1S1B2I1 C2S2B2I2 C1S1B2I1 C1S1B3I1 C1S1B1I1 C1S1BI11 C1S1B2I1 C1S1B2I1 C2S1B2I1
**Hemodynamic (10)**								
Discomfort, hypotension Discomfort, dizziness 3 Syncope Dizziness, myosis 2 Discomfort Discomfort, loss of consciousness Hypotension, vomiting	F27 M23 M79 F82 F35 F28 M80 F8 F3 M63	NA Allergy NA NA NA NA NA Anorexia NA Anorexia	NA 8 12 8 12 4 8 2 4 4	NA 1 30 12 21 1 NA 60 30 1	prazepam, milnacipran N Pipemidic acid, ambroxol, temazepam, codeine, DEC zolpidem, tianeptine, clomipramine, lorazepam ergocalciferol, lorazepam N nicergoline, amiodarone, buflomedil, hawthorn citrulline malate, captodiamine N N	Y Y NA NA NA Y NA NA NA NA	Y (NA) Y (3) Y (NA) Y (NA) NA (NA) Y (NA) NA (NA) Y (NA) Y (NA) Y (1)	C1S1B1I1 C2S1B3I1 C2S1B3I1 C2S2B3I2 C1S2B1I1 C3S1B2I3 C1S2B3I1 C2S1B2I1 C3S1B3I3 C1S1B2I1
**Haemaotologic (10)**								
Anemia and neutropenia 2 Neutropenia Thrombopenia 4 Anemia Hypereosinophilia Pancytopenia 1	F77 M71 F18 F77 F85 F92 F82 M81 M84 M63	Scab NA Anorexia NA NA NA NA NA Orexigenic Allergy	12 12 4 NA NA 8 NA 4 4 12	NA 12 15 NA NA NA 60 15 5 7	dexchlopheniramine, pseudoephedrine pefloxacin etybenzatropine, chlorpromazine, sulpiride heparin, gentamicin, pefloxacin lidocaine, spironolactone N amineptine, flunitrazepam, lisinopril, nicardipine, furosemide DES, furosemide, prednisone, omeprazole, clopidogrel noramidopyrine-caffeine, lactitol carboplatin, pemetrexed	Y Y Y NA Y Y Y NA Y Y	Y (11) Y (NA) Y (7) Y (NA) Y (NA) NA (NA) Y (NA) N (17) Y (7) Y (NA)	C2S1B3I1 C3S2B3I3 C1S1B2I1 C1S1B2I1 C1S1B1I1 C2S2B3I2 C2S1B3I1 C1S1B2I1 C2S1B1I1 C2S1B2I1
**Dermatologic (8)**								
Rash 6 Erytheme polymorphe Stevens Johnson	F78 F60 F32 M5 F33 M67 M93 M30	NA NA Allergy NA NA NA NA NA	12 12 8 4 24 8 12 NA	3 4 8 4 17 4 150 30	N vinorelbine tetracycline, tritoqualine N N rifampicin pinaverium bromide, pancreatic extracts, loperamide maprotiline, fluoxetine	Y Y N NA Y NA NA Y	Y (NA) Y (NA) Y (NA) Y (NA) Y (NA) Y (NA) NA (NA) Y (5)	C2S2B3I2 C2S1B3I1 C1S1B2I1 C2S1B3I1 C2S1B2I1 C2S2B3I2 C1S1B1I1 C2S1B1I1
**Miscellaneous (12)**								
Glaucoma 1 Gynecomastia 1 Diarrhea 2 Urine retention 4 Hypothyroidia 1 Anxiety 1 Pharmacodependance 1 Renal acute failure and rhabdomyolyse 1	F81 M30 M78 F80 F34 M99 F2 M79 M80 F21 M37 M60	NA Orexigenic NA NA NA NA NA Anorexia NA Anorexia Allergy NA	4 NA 4 8 24 4 4 12 NA NA 16 NA	NA 30 3 30 4 9 90 4 A NA 730 150	clomipramine N bromazepam, hydroquinidine chlorhydrate N N N N N amiodarone, dipyridamole, theophylline N N flunarizine, magnesium, mebeverine, colimycin	NA Y NA Y NA Y Y Y NA Y Y NA	Sequelae (NA) N (15) Y (NA) Y (NA) Y (NA) Y (NA) Y (NA) Y (1) NA (NA) (NA) NA Y (NA)	C2S1B2I1 C1S1B1I1 C1S2B3I1 C3S2B3I3 C2S1B1I1 C1S1B3I1 C1S1B3I1 C2S1B3I1 C1S1B3I1 C2S1I1 C3S3B2I4 C2S1B0I1

*CY, cyproheptadine; DEC, dihydroergocristine; DES, diethylstilbestrol; DXP, dextropropoxyphene; EE, ethinylestradiol; F, female; GGT, gamma-glutamyl transferase; M, male; N, no; NA, not available; PCT, paracetamol; UR, unspecified reason; SAP, serum alkaline phosphatase; Y, yes*.

The median dosage observed was 8 mg per day (range 4–24 mg) in adults, and 4 mg per day (range 2–20 mg) in children. In France before the 1990s, the maximum recommended dosage was 20 mg per day for adults, 16 mg per day for children aged of 7 to 14 years old, and 12 mg per day for children aged of 2–6 years old. In the 2000s, the usual dosage was 20 mg per day for adults and 12 mg per day for children older than 6 years. Subsequently, the median dosage we observed was consistent with recommendations, and was higher in only four cases.

**Figure 2:**
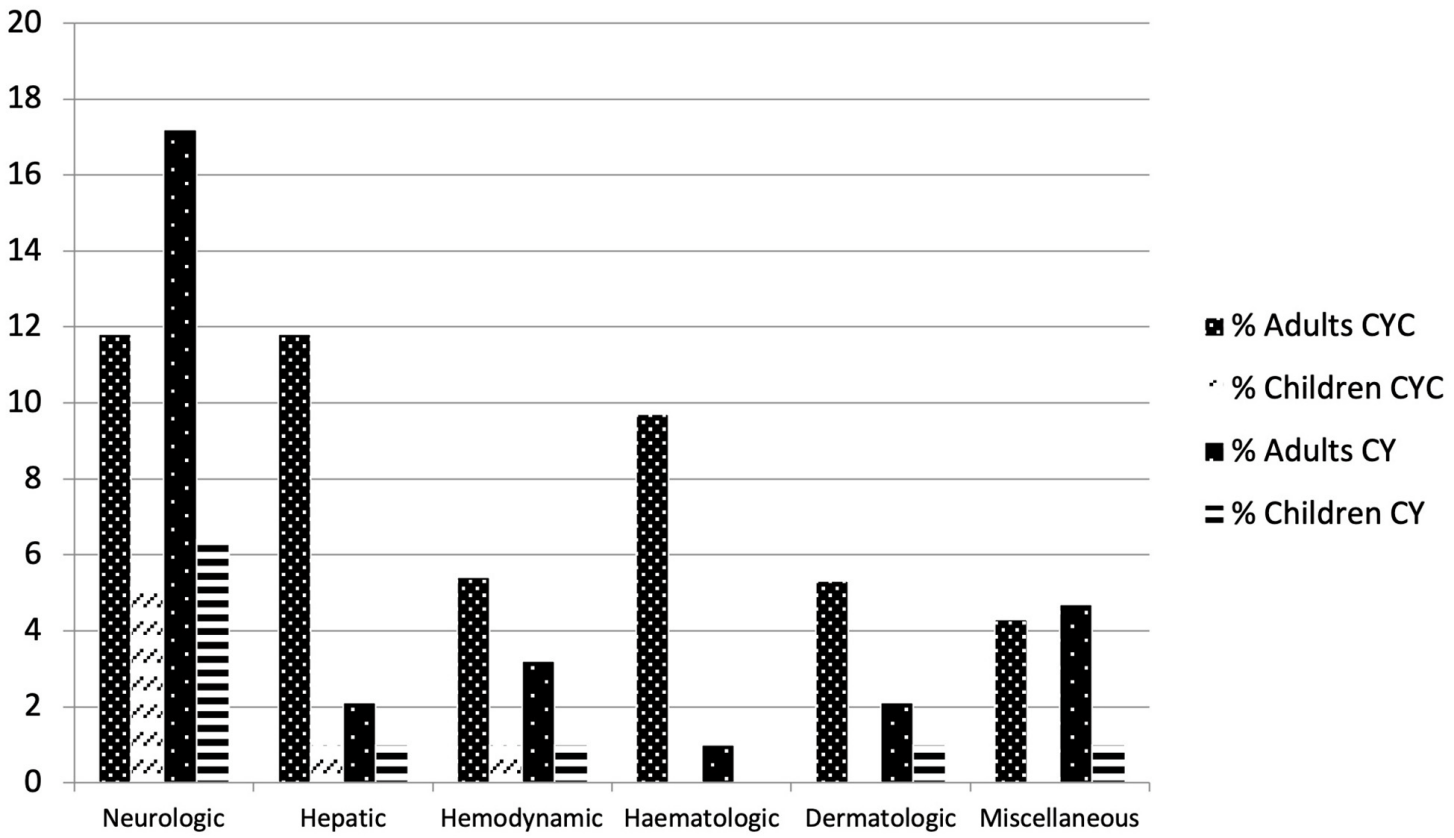
Cyproheptadine adverses effects in adults or children in the French pharmacoviligance database, with cyproheptadine (CY) as monotherapy or with concomitant medications (CYC) (data are percentage of 93 AEs).

The median delay between the start of cyproheptadine treatment and the AE occurrence was 10 days (range 1–180 days). Among the 93 AEs, 40.8% were neurological symptoms (*n* = 38 including seven drowsiness, six confusion, five seizures, five agitation, five hallucinations, two asthenias, two paresthesias, and miscellaneous), 16.1% were hepatic lesions (*n* = 15), 10.7% were hemodynamic troubles (*n* = 10), 10.7% were hematological features (*n* = 10), 8.6% were dermatological symptoms (*n* = 8), 4.3% were urine retentions (*n* = 4), 2.1% were diarrheas (*n* = 2), and some miscellaneous (1 glaucoma, 1 hypothyroidism, 1 gynecomastia, 1 rhabdomyolysis). The most severe AEs were liver failures (*n* = 3), and central nervous system symptoms. Among all patients, 36 patients received cyproheptadine as a monotherapy, and 57 had a suspected concomitant treatment. Patients treated with cyproheptadine in monotherapy mainly had neurological symptoms, although one adult and one child had liver failure (Figure 2).

We analyzed more thoroughly the 15 reported cases of hepatic complications. Among the three patients who had cyproheptadine as monotherapy, a 1-year-old child died from acute liver failure (but a syphilitic infection was suspected at autopsy), one adult presented moderate cytolysis, and one adult had hepatic failure that resolved. Twelve patients received cyproheptadine with other suspected concomitant drugs: among them, most had moderate hepatitis (*n* = 6), or cholestasis (*n* = 3) that resolved with cyproheptadine withdrawal, one child had acute liver failure (hepatitis B, evolution not available), and two patients had high serum alkaline phosphatase level or high gamma-glutamyl transferase levels (evolution not available).

For the 93 patients, when specified, cyproheptadine was always discontinued (*n* = 57). The resolution of the AE was specified for 80 patients: 91.2% of patients had total resolution (*n* = 73), 1.2% had partial resolution (*n* = 1), 3.7% had no resolution (*n* = 3), and 3.7% of patients died (the child with suspected syphilitic infection, and two adults died from a cause other other than cyproheptadine). According to the French causality assessment, the cyproheptadine imputability score for all 93 AEs ranged from 1 to 4 (score 1 *n* = 68, score 2 *n* = 18, score 3 *n* = 6, score 4 *n* = 1). Specifically, scores were of 1 or 2 for hepatic AEs.

We next sought to estimate the frequency of the AEs in France during the period studied. OpenHealth informed us that 2,169,221 boxes had been sold between January 2008 and December 2020, with a median of 164,054 ± 8,588 boxes were sold per year, and this number being quite stable per year. Since OpenHealth collects data on drugs sales from approximately half of the retail pharmacies in France, we could extrapolate that about 328,108 boxes were sold per year in France. A box contains 30 tablets (4 mg per tablet). Considering that a patient takes 8 mg per day on average, representing 24 boxes per year, we estimated that more than 13,672 patients took a cyproheptadine medication each year in France. Considering that the boxes sold per year were the same before 2008, we estimated that the 93 AEs occurred in more than 13,672 patients between 1985 and December 2020, which represented a frequency lower than 0.7% (7 AEs for 1,000 patients). For hepatic AEs, the frequency was about 1/1,000 patients, which is considered as an uncommon AE, according to the international classification of medication AEs (very common is ≥ 1/10, common is ≥ 1/100 to <1/10, uncommon is ≥ 1/1,000 to <1/100, rare is ≥1/10,000 to <1/1,000, and very rare is <1/10,000). Using the same method, the estimated frequency in this database was 0.3 % for neurological symptoms, 0.07% for hemodynamic symptoms, 0.07% for hematological symptoms and 0.05% for dermatological symptoms. Conversely, we also considered that a patient could take 8 mg per day for 3 months, representing 6 boxes par year, which would represent more than 54,685 patients taking cyproheptadine per year. Consequently, the estimated frequency of all AEs would be lower than 0.17%, and that of hepatic AEs would be rare, at 0.27/1,000. As such, we estimated that the frequency of hepatic AEs with cyproheptadine was probably between 0.27 and 1/1,000 in France during this period.

### Literature Systematic Review of Adverse Events of Cyproheptadine

Among 8,602 articles, we selected 171 fulls text articles, which included case reports (*n* = 72), randomized controlled trials (*n* = 39), prospective trials (*n* = 51) and retrospective trials (*n* = 9) (Figure 1). Overall, 105 articles concerned adults, and 66 concerned children, which represented a total of 3,478 patients. A few studies included infants (16, 17, 22). All reports were published between 1960 and 2020, and most (74%) before year 2000. The indications for cyproheptadine therapy varied greatly as described in Table 3. The duration of treatment was heterogenous and differed according to the indication for cyproheptadine: from a single dose to extended treatment, and mainly for orexigenic effects (median duration 56 days, range 1–870). The longuest duration was of 29 months described in a pediatric case report (23).

**Table 3:** Indications for cyproheptadine use in the PRISMA-literature review.

**Indications for cyproheptadine use**	**Publications *n* (%)**
Orexigenic effect	45 (26.3)
Endocrinal diseases (Cushing disease, Nelson syndrome, hypopituitarism, acromegalia, hyperparathyroidism)	31 (18.1)
Neuropsychic diseases (autism, schizophrenia, neuroleptic adverse events prophylaxis, nightmares, attention deficit hyperactivity disorder)	14 (8.2)
Dermatological (urticarial, prurit, mastocytosis, acanthosis nigricans)	14 (8.2)
Accidental	13 (7.6)
Anorgasmia	10 (5.8)
Experimental studies in healthy adults or children subjects	8 (4.7)
Functional digestive disorders (abdominal recurrent pain, cyclic vomiting syndrome, dyspepsia)	7 (4)
Muscular diseases	6 (3.5)
Migraine prophylaxis	5 (2.9)
Carcinoid syndrome	5 (2.9)
Serotoninergic syndrome	5 (2.9)
Miscellaneous: Prinzmetal angina, parasitological diseases, blepharospasm, cerebral vasoconstriction syndrome	5 (2.9)
Allergic diseases (allergic rhinitis, hay fever, asthma)	3 (1.7)

Among these 171 articles, 61.4% reported some AEs, in adults (*n* = 53), or children (*n* = 52). The median cyproheptadine dosage did not differ whether reporting AEs or not: it was 12 mg per day for adults (range 2–37.5), and 0.25 mg/kg/day (range 0.1–0.8), or 7.5 mg/day (range 1–16) for children. These dosages were consistent with the French, Canadian and US recommandations (24–26). All AEs appeared within a few days after that start of cyproheptadine treatment.

For the 3,478 patients who received cyproheptadine, the exact number of patients affected by an AE was not always specified in the report, although the most frequent AE reported in publications was drowsiness (Table 4). In randomized controlled trials, drowsiness was significant in a large trial including 295 adults (15), and in a small trial (27), but was not in other trials (28–30). Weight gain and increased appetite were also reported as adverse or beneficial effects, depending on the purpose of the study. Other AEs were more rarely reported. When cyproheptadine was used to treat serotonine syndrome, it was generally well-tolerated and efficient, although tachycardia, sedation, hyperthermia, delirium, urinary retention, dilated pupils, decreased bowel movements, dry mouth and dry skin were described (31). No dermatologic or haematologic AEs were reported.

**Table 4:** Adverse effects (AEs) with cyproheptadine (overdose cases excluded) reported in the literature.

**Type of AEs**	**Number of publications citing this AE**	**Number of AE cases**	**References**
Drowsiness	*n* = 59	NA (*n* = 418 cases notified in 49 publications, NA in others)	(1, 2, 5, 7, 8, 15–17, 19, 22, 27–75)
Weight gain or increased appetite	*n* = 52	NA (*n* = 755 cases notified in 46 publications, NA in others)	(1, 2, 5–7, 12, 15–17, 22, 28, 29, 32, 34, 35, 37, 39–42, 44, 48, 49, 52–54, 56, 57, 60, 62, 63, 66, 67, 69–71, 73–88)
Dry mouth or nasal mucosae	*n* = 11	NA (*n =* 80 cases notified in 7 publications, NA in others)	(5, 8, 15, 31, 42, 60, 66, 70, 72, 73, 85, 89)
Hepatic complications	*n* = 5	5	(18, 90–93) (in Table 5)
Irritability	*n* = 4	18	(6, 16, 17, 61)
Headache	*n* = 6	NA (*n =* 17 cases in 3 publications, NA in others)	(5, 41, 61, 73, 79, 94)
Dizziness	*n =* 5	NA (*n =* 38 cases notified in 2 publications, NA in others)	(15, 60, 61, 66, 70)
Agitation	*n =* 4	4	(2, 29, 37, 95)
Nauseas	*n =* 3	NA (*n =* 48 in 2 publications, NA in others)	(15, 61, 94)
Insomnia, sleep disturbance	*n =* 3	NA (*n =* 9 in 1 publication, NA in others)	(5, 8, 73)
Constipation	*n =* 3	9	(22, 31, 34)
Hallucinations, delirium	*n =* 3	7	(31, 96, 97)
Acute urine retention	*n =* 3	4	(31, 98, 99)
Behavioral changes	*n =* 2	7	(16, 22)
Diarrhea	*n =* 2	3	(2, 34)
Anticholinergic syndrome	*n =* 2	2	(89, 97)
Blurred vision	*n =* 2	NA (*n =* 1 in 1 publication, NA in 1 other)	(60, 72)
Vomiting	*n =* 2	34	(15, 60)
Excess virilization	*n =* 2	5	(73, 100)
Swallowing troubles	*n =* 1	2	(34)
Abdominal pain	*n =* 1	2	(16)
Stiffness	*n =* 1	1	(34)
Toxic psychosis	*n =* 1	1	(101)
Obsessive compulsive troubles	*n =* 1	1	(102)
Facial oedema	*n =* 1	1	(42)
Nightmare	*n =* 1	1	(103)
Slow movement	*n =* 1	1	(34)
Recurrence of depression	*n =* 1	1	(104)
Dilated pupils	*n =* 1	2	(31)
Hyperthermia	*n =* 1	5	(31)
Tachycardia	*n =* 1	13	(31)
Serotonin syndrome after cyproheptadine withdrawal	*n =* 1	1	(105)

*NA not available (=authors did not give the exact AE number in the publication)*.

Five case reports describing hepatic complications were published between 1971 and 2014 (Table 5): these included four cholestatic hepatitis cases and one acute liver failure case which occured 5 to 35 days after start of cyproheptadine treatment. All patients had a favorable evolution after cyproheptadine withdrawal. No patients had any prior history of liver disease. In all other publications, hepatic blood tests were rarely performed. Only two publications reported hepatic blood tests which were normal (32), or showed isolated high serum alkaline phosphatase levels (76). Our systematic review of the literature found that hepatic complications with cyproheptadine treatment occurred in 1.4/1,000 patients (5 cases among 3,478 patients), and could be considered as an uncommon AE according to the international classification of medication AEs.

**Table 5:** Case reports of hepatic adverse events (AEs) with cyproheptadine reported in literature.

**Publications**	**Type of AEs**	**Patients** **(Sex/age, years)**	**Indication**	**Dosage (mg/ day)**	**Delay after introduction**	**Concomitant suspect medication**	**cyproheptadine discontinuation**	**Resolution (duration of follow-up)**
Karkalas and Lai (92)	Cholestatic hepatitis	1 adult (M59)	psoriasis prurit	16	5 weeks	Imipramine	Y	Y (3 weeks)
Henry et al. (93)	Cholestatic hepatitis	1 adult (F25)	prurit	12	1 month	None	Y	Y (2 months)
Larrey et al. (90)	Cholestatic hepatitis	1 adult (NA)	anorexia nervosa	12	5 days	acetylsalicylic acid ethinylestradiol, quingestrone	Y	Y (3 weeks for ALT) GGT still high at 31 months
Freneaux et al. (91)	Cholestatic hepatitis	1 adult (F23)	orexigenic	8	1 month	dihydroergocristine magnesium + pyridoxine methionine + cysteine	Y	Y (3 months)
Chertoff et al. (18)	Acute liver failure (and kidney injury)	1 adult (F55)	orexigenic	NA	3 weeks	none	Y	Y (3 weeks)

*ALT, alanine aminotransferase; F, female; GGT, gamma-glutamyl transferase; M, male; NA, not available; Y, yes*.

In cases with overdoses (*n* = 91), patients presented mostly with anticholinergic syndrome, within hours of cyproheptadine ingestion (19, 106–113), and with periphereal and/or central nervous system manifestations, including two deaths in adults (114, 115). Blood hepatic tests were rarely performed in these situations; in two cases, these were normal (116, 117).

## Discussion

Our analysis of the cases reported in both the French pharmacovigilance database and the literature confirms that cyproheptadine is a safe drug, although physicians should be aware of potential severe hepatic complications. Also, AEs in infants may not yet be well-known due to a lack of studies in this age group. While not all side effects may have been recorded in this database or been published, it is likely that the most severe cases have been reported. Indeed, the cases recorded in the French national pharmacovigilance database are based on voluntary reports from physicians. We could not calculate a precise prevalence or risk of AEs with cyproheptadine because we do not know the exact number of AEs, of ingested cyproheptadine tablets and people who took the drug. However, it is interesting to note that the number of boxes sold per year in France has been quite stable between 2008 and 2020. This suggests that cyproheptadine is currently used mainly for its orexigenic properties, since its indication for allergy relief has been supplanted by newer-generation anti-H1 drugs.

As described in previous studies, the most frequent AEs were mild neurological complications such as drowsiness, dizziness, confusion, and agitation as all first generation H1-antagonists cross the blood-brain barrier. The AEs can be explained by cyproheptadine's antihistaminic properties (drowsiness, discomfort), anticholinergic properties with periphereal symptoms (urinary retention, tachycardia, facial flushing, hyperpyrexia, dry mucous membranes, dilated pupils, constipation) or central symptoms (dizziness, confusion, agitation seizures, athetosis, hallucination, delirium), antiadrenergic properties (orthostatic hypotension, dizziness), and antiserotoninergic properties (weight gain, augmentation of appetite). The responsibility of cyproheptadine in relation to rash, haematologic AEs, gynecomastia, and diarrhea is more doubtful: such AEs were rarely reported, and in our database, concomitant medications could have induced these effects. All of these AEs disappeared after cyproheptadine discontinuation. In *in vivo* and *in vitro* studies, cyproheptadine did not induce cardiovascular AE complications (118), which were reported mainly with other H1-antagonists such as diphenhydramine and hydroxyzine (14).

Of more concern and less well-known are hepatic complications associated with cyproheptadine, which may be severe. A total of 15 cases were recorded in our database between 1986 and 2016 and five cases were found in the literature, affecting two children and 18 adults. We estimated the frequency of hepatic AEs to be of 0.27 to 1.4/1,000 (uncommon to rare). Because patients are usually not monitored by hepatic blood tests, we cannot exclude that hepatic perturbations are underdiagnosed. When follow-up data was available, we observed that moderate cytolysis and cholestatic hepatitis resolved in 1–3 weeks and in 3 weeks to 8 months, respectively, after cyproheptadine withdrawal. As four patients had acute liver failure, including two without concomitant medications or other possible etiology, cyproheptadine should probably be contraindicated in patients with prior liver disease. For other patients, hepatic blood test monitoring should be initiated in future trials to screen for this potential complication.

Cyproheptadine hepatotoxicity could be due to its structure (tricyclic ring), which is similar to phenothiazine drugs (18, 90). The structure also contains a tertiary amine that could induce decoupling properties of oxidative phosphorylation (119). In addition, an immunoallergic process has been suspected, and in one case, hypereosinophilia was associated with the hepatic event (91). In an experimental study, rats treated with cyproheptadine had significantly elevated of hepatic microsomal cytochrome P450 levels and ultrastructural alterations to liver cells, suggesting a certain degree of hepatotoxicity with cyproheptadine (120). Accordingly, cyproheptadine is considered a potential hepatotoxic drug (121), classified as category C in LiverTox, and a probable rare cause of clinically apparent liver injury (122). The gold standard of the diagnosis of drug-induced liver injury is the recurrence of liver test abnormalities upon readministration of the drug, although in practice this is rarely done (123). Hepatic complications have also been described with second-generation H1-antihistamines: loratadine or desloratadine (124, 125), cetirizine (126), and terfenadine (127), with good evolution after drug's discontinuation.

There are very few studies evaluating infants treated with cyproheptadine. In the United States, cyproheptadine is contraindicated in infants, “because a paradoxical central nervous system stimulation and/or respiratory depression can occur,” according to the Prescribers Digital Reference (26). These recommendations are related to the reports of respiratory depression, sleep apnea, and sudden infant death syndrome in children that received phenothiazine drugs, which share a similar structure with cyproheptadine. We did not observe such AEs in our database or our literature review, although few studies included infants (16, 17, 22).

In summary, the reported AEs with cyproheptadine treatment in the French pharmacovigilance database and in the literature support the idea that cyproheptadine can be considered as a safe drug. We found that mild neurological effects were frequent, and that hepatotoxicity was uncommon to rare. However, randomized controlled trials are still needed, in terms of safety and efficacy, in order to modify the authorization of cyproheptadine for appetite stimulation, especially in young children and infants for whom studies are lacking. The prescription of cyproheptadine must follow the principles of estimating the benefit/risk ratio for each patient and should respect the classical dosage for an orexigenic indication (0.25 mg/kg/day for children, 8–12 mg/day for adults). Cyproheptadine should not be prescribed in patients with prior liver disease, and possible hepatic complications should be monitored in future trials as these may have been underdiagnosed.

## Data Availability Statement

The original contributions generated for the study are included in the article/supplementary material, further inquiries can be directed to the corresponding author/s.

## Author Contributions

VB conceptualized and designed the study, collected and analyzed data, performed the literature search, drafted the initial manuscript, and revised the manuscript. NM collected data, contributed to analyzing data, interpreting results, and reviewed and revised the manuscript. NV, VG, and CC contributed to analyzing data, interpreting results, and reviewed and revised the manuscript. M-PT contributed to analyzing data, and reviewed and revised the manuscript. VA conceptualized and designed the study, analyzed data, performed the literature search, reviewed and revised the manuscript. All authors contributed to the article and approved the submitted version.

## Conflict of Interest

The authors declare that the research was conducted in the absence of any commercial or financial relationships that could be construed as a potential conflict of interest.

## Publisher's Note

All claims expressed in this article are solely those of the authors and do not necessarily represent those of their affiliated organizations, or those of the publisher, the editors and the reviewers. Any product that may be evaluated in this article, or claim that may be made by its manufacturer, is not guaranteed or endorsed by the publisher.

## Abbreviations

AEs: adverse effects.
